# Memorials to Dr WG Grace – general practitioner and cricketing legend

**DOI:** 10.1177/09677720241227420

**Published:** 2024-03-13

**Authors:** Neil G Snowise

**Affiliations:** 405987 Centre for Pharmaceutical Medicine Research, Institute of Pharmaceutical Science, King's College, London, UK

**Keywords:** Legacy, memorials, general practice, cricket, commemorations

## Abstract

Dr WG Grace was a general practitioner in Bristol, in the late nineteenth century, but is better remembered as ‘the father of cricket’. He showed early promise as a skilled cricket player and was already playing for Gloucester County, by the age of fifteen. However, coming from a well-established medical family, his father wanted him to become a doctor. He trained in Bristol and after qualifying he set up his own practice in the same environs. By this time, he was a superb cricketer with a glittering county and England career, combined with his clinical duties. He has several memorials where he lived and practised in Bristol, which are described and illustrated in this review. These include commemorative plaques in the local church, and near his later residence in Clifton, as well as a large mural at a train station and another at a shopping centre. These are all tributes to one of the most famous sons of Bristol. He is also celebrated at Lord's Cricket Ground, the home of cricket, with eponymous memorial gates and a full-size statue inside the ground. A fine example of a doctor who also had other talents, these memorials reflect his widespread appeal and his long-lasting legacy.

Dr William Gilbert ‘WG’ Grace (1848–1915) is the most famous cricketing doctor and Bristol's most famous sportsman. He was a pioneer of cricket, helping to bring it to public attention, by a combination of his cricketing ability and, in no small part, by his larger-than-life personality. WG Grace was a showman and a sporting celebrity of his era who attracted huge crowds to the game.

Universally known by several nicknames including ‘WG’ or ‘The Doctor’, he was an outstanding all-rounder despite being best remembered as a batsman (Figure 1). Much has been written about his life,^1,2^ including an account in the first volume of this journal 30 years ago.^3^ This review highlights some of the varied memorials that commemorate his life; some have been in the same place for many years, while others were not in existence 30 years ago when this journal was first published.

**Figure 1.**
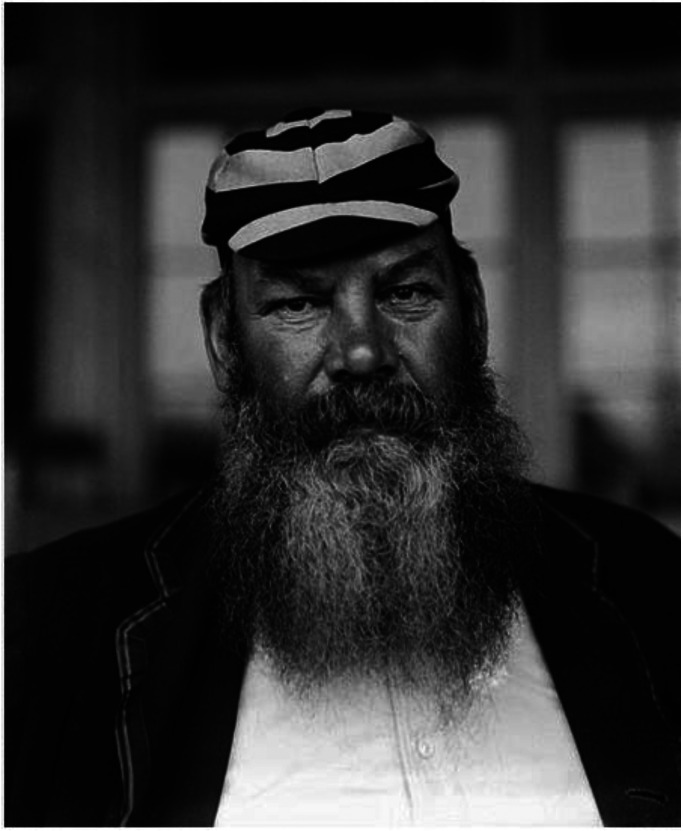
Dr WG Grace 1848–1915 with his trademark beard.

## Early life in Bristol

One of nine children, he was born in Downend which at the time was described as ‘a small Gloucestershire village’,^1^ but is clearly now a Bristol suburb. He came from a very well-established medical family – he was the fourth son of Dr Henry Mills Grace, who was a general practitioner in Downend, and his three older brothers all became doctors. The eldest son, Henry, and the second son, Alfred, were general practitioners while the third son, Edward, became the medically qualified coroner for the Lower Division of Gloucestershire.

**Figure 2.**
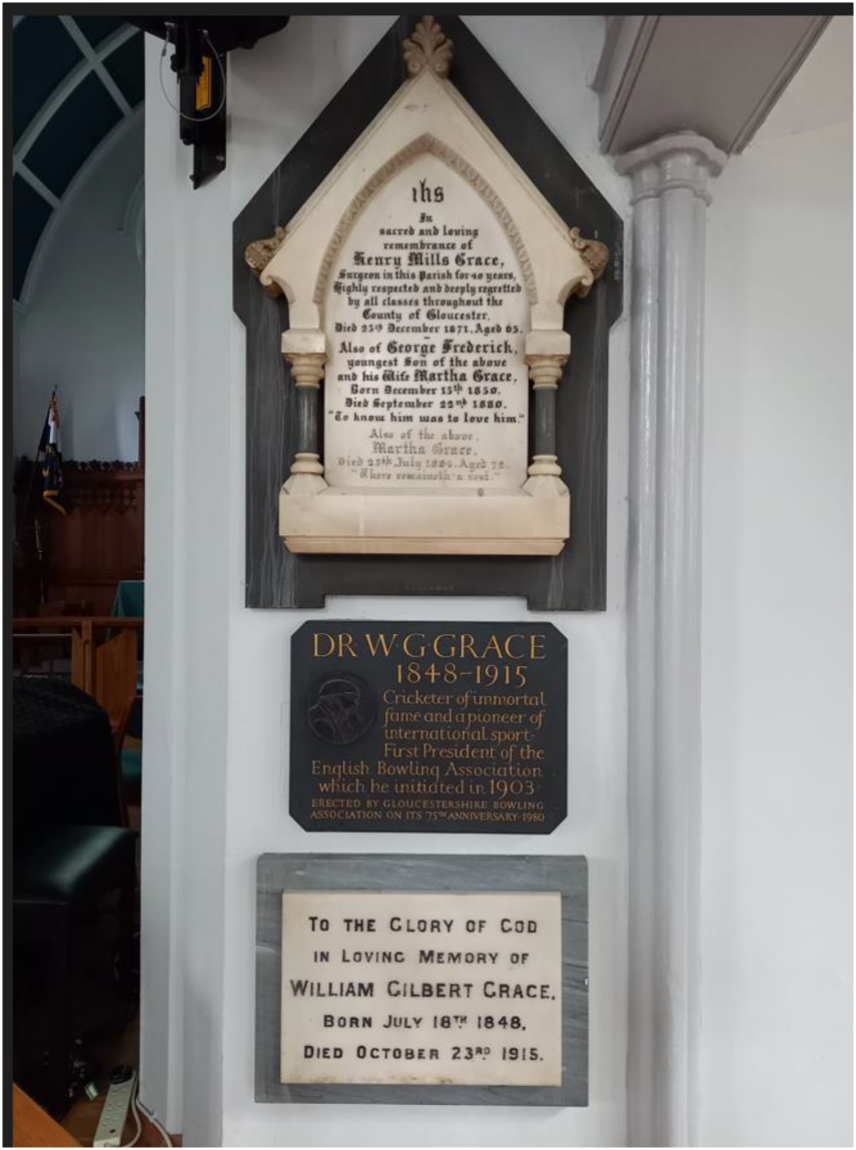
Commemorative plaques to the Grace family in Christ Church, Downend, Bristol.

He was baptised at Christ Church in Downend, which has several commemorative plaques for the Grace family (Figure 2). His father Henry, mother Martha and youngest brother George, who sadly died from pneumonia at age 30, are remembered in the top elaborate plaque. Henry was described in the inscription as ‘surgeon’ and ‘highly respected … by all classes’, reflecting his medical practice in a poor area of Bristol, with varied social classes living there. The surrounding neighbourhood had its fair share of paupers, but most of Grace's patients had the money to pay him fees. Underneath there are two commemorative plaques dedicated to WG Grace.

Grace appears to be the most famous son of Downend and there are several memorials that remain in existence there. Christ Church overlooks the adjacent Downend Cricket Club, where the Pavilion was erected in his memory in 1922. Sixty years later a large mural of Grace was created and placed on the wall above the entrance of a new shopping mall nearby,^4^ which is still in situ today.

## Cricketing talent at an early age

Encouraged and coached by his father and older brothers, his cricketing prowess emerged early. By age 15, he was already playing for Gloucester County and was chosen to play for the ‘Gentlemen (amateurs) versus Players (professionals)’ match. Before the start of Test cricket in 1877, Gentlemen versus Players was the most prestigious cricket fixture in which a player could take part. While still a teenager, he was playing regular first-class cricket and scored his first-ever century in the match against ‘Gentlemen of Sussex’. Just after his 18th birthday in July 1866, Grace confirmed his potential with an innings of 224 for All-England against Surrey at The Oval.

However, his father had other ambitions for him and wanted him to become a doctor. He started at Bristol Medical School in 1868, taking until 1879 to qualify because cricket had become his undoubted priority. His academic work (both performance and attendance) could be described as inversely proportional to his dedication to playing cricket, as he had some of his best seasons during those years, impressing everyone with his cricketing skills.

In 1869, Grace became a member of the famous Marylebone Cricket Club (MCC). In the same year, he scored four centuries in the summer, including an innings of 180 at The Oval, Surrey. This was achieved in the opening wicket partnership of 283 with Bransby Cooper and was Grace's highest partnership total of his career.

Grace was married in 1873 to Agnes Nicholls Day, who was the daughter of his first cousin William Day. Two weeks later, they began their honeymoon by travelling to Australia for Grace's 1873–74 tour. Effectively this was an England side, but the tour was before Test matches commenced and the team was known as the WG Grace XI.

They returned from the ‘cricketing honeymoon’ tour in May 1874 with Agnes 6 months pregnant.

The Graces moved to London in February 1875, when he was attached to St Bartholomew's Hospital, to continue his clinical studies. A ward in the Queen Elizabeth II Wing at St Bartholomew's Hospital used to bear the name ‘WG Grace Ward’ until the building was demolished.

## Combining medical practice with cricket

There was speculation that Grace intended to retire before the 1878 season to concentrate on his medical career, but he decided to continue playing cricket; his decision may have been influenced by the arrival of the first Australian team to tour England in May. Following the 1878 season, Grace was assigned to Westminster Hospital Medical School for his final year of medical practice and this curtailed his cricket for a time as he did not play in the 1879 season until June.

However, the upheaval was worthwhile because, in 1879, Grace finally qualified as a Licentiate of the Royal College of Physicians (Edinburgh) and a Member of the Royal College of Surgeons (England). Although he had to prioritise his clinical practice for a few years in his new position, he still found time to make his first appearance in Test cricket the following year. He scored the first-ever century in Test cricket by an England batsman, in 1880, against Australia. Overall, he played for England in 22 Tests through the 1880s and 1890s, all of them against Australia.

Grace was not without controversy. Instantly recognisable at six feet, two inches tall with his trademark beard and ‘ample girth’, he was no shrinking violet. As a member of the medical profession, he was nominally an amateur (a gentleman) cricketer, not a paid professional (a player), but he is said to have made more money from his cricketing activities than any professional, which could lead to some hostility from the other players. In 1879, he had to answer charges of claiming exorbitant expenses; he was forced to liaise in the future with a new finance committee and abide by stricter rules.

**Figure 3.**
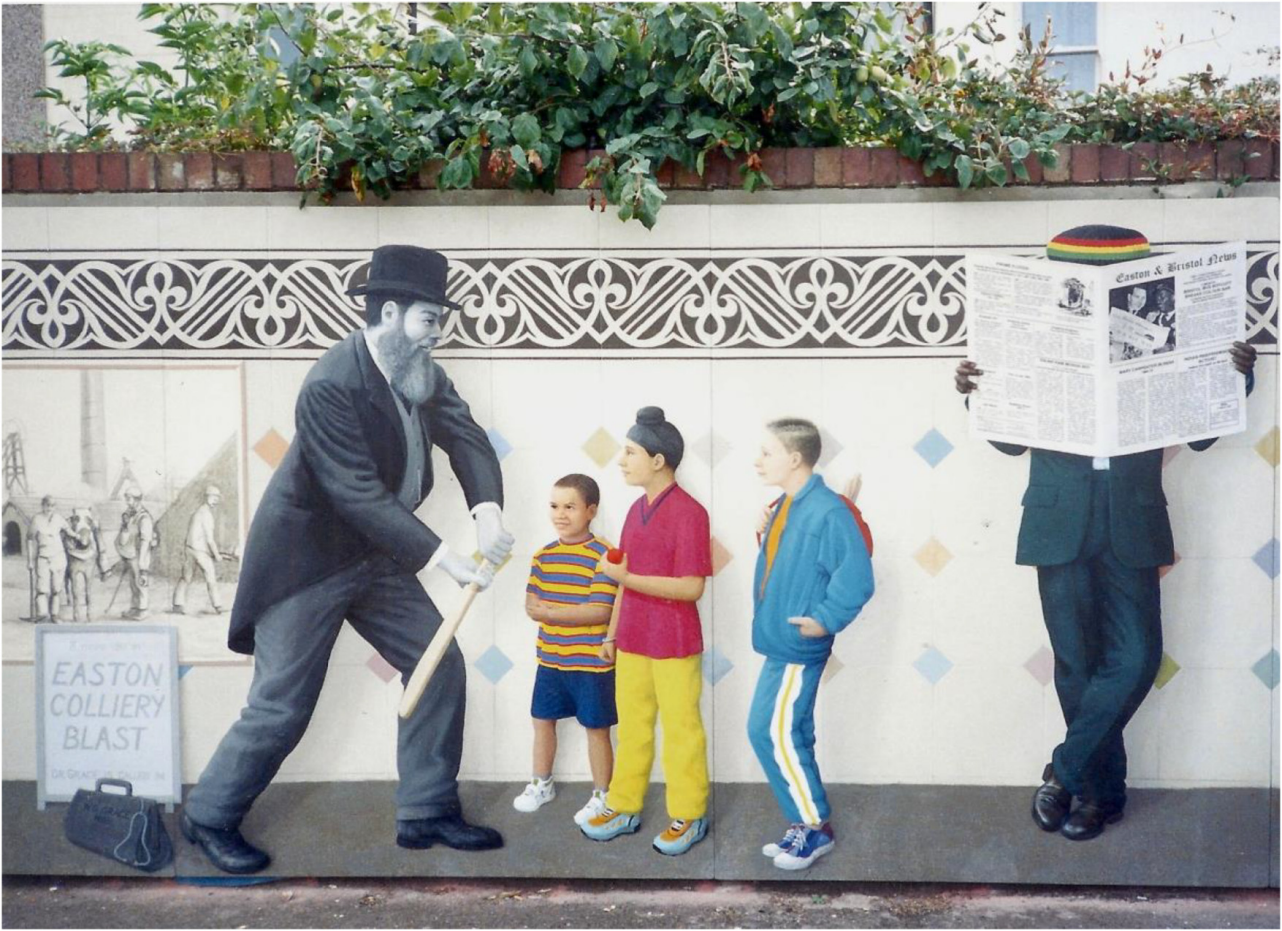
WG Grace pictured at Stapleton Road station, Bristol.

After qualifying he worked in his own practice at Stapleton Road in Easton, a largely poor district of Bristol, employing two locums during the cricket season. He undertook other medical duties outside his practice – he was the local public vaccinator and was the medical officer of the Barton Regis Union. Stapleton Road train station is on a railway line from the centre of Bristol, where there is an interesting mural, created in 1999 (Figure 3).

As part of the Easton Renewal Area Project, the 25 m-long platform mural is a portrait of the local community and features 30 life-size figures waiting for a train.^5^ Historical figures are painted in black and white, while modern characters are in colour. Grace is featured, coaching some modern-day children, as well as paintings of some other famous local residents – Raj Ramohan Roy, 19th-century social reformer, and Ben Tillett, Trade Union leader. His medical bag is on his left-hand side, with the notice ‘Dr Grace is called in’, referring to him treating casualties at a local colliery accident, which sadly led to eight deaths.^6^ The newspaper on the right has specially written text, including a short column about Grace. Originally painted in 1999, the mural weathered over the years and was fully restored in 2015.^7^

**Figure 4.**
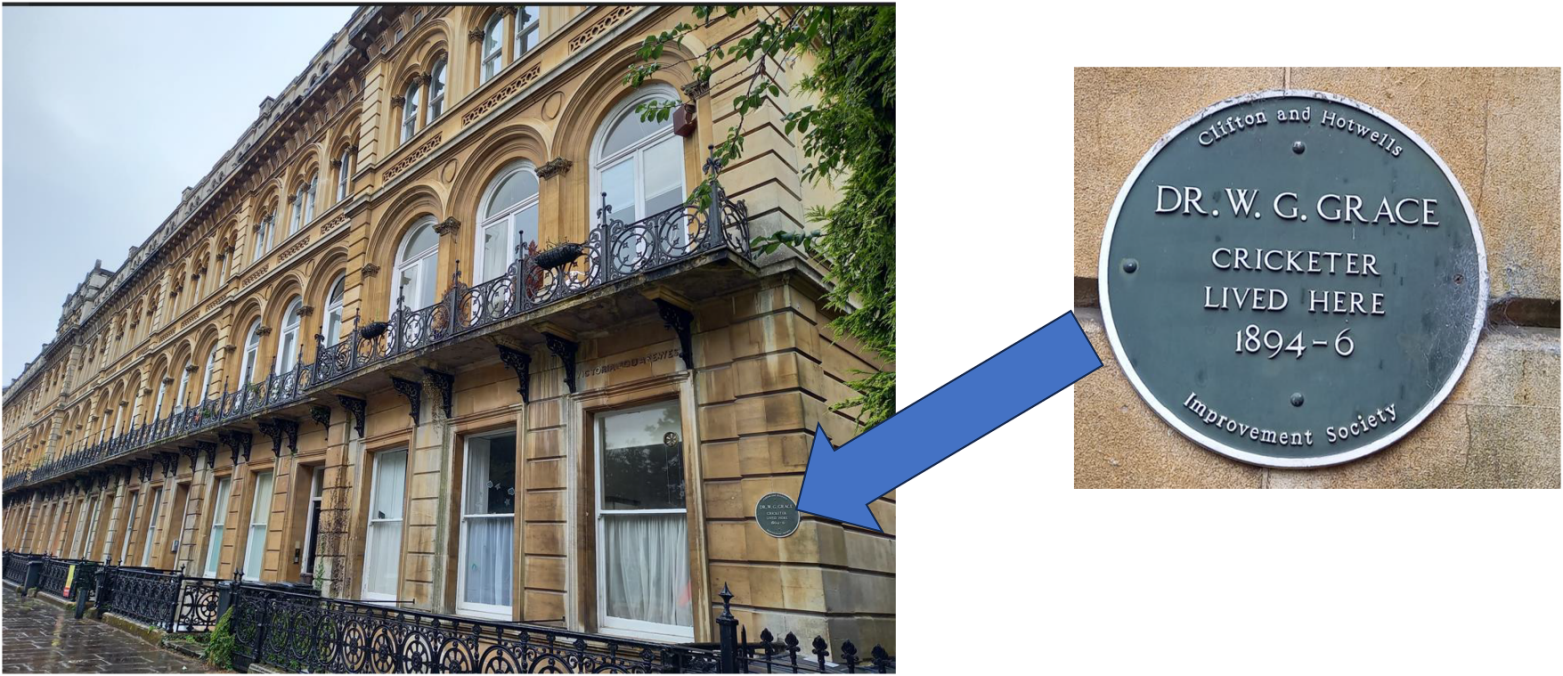
Memorial plaque in Clifton, Bristol.

He moved house several times, whilst in medical practice in Bristol. He lived in more fashionable Clifton in the 1890s, where he is remembered with a plaque, in the elegant Victoria Square, where he lived at number 15. This was erected by the Clifton and Hotwells Improvement Society, in 1992 (Figure 4).

There are also two public houses named after him in Bristol, although there is little evidence of Grace memorabilia on the premises. More recently, in 2021, a commemorative bronze bust went on display outside the Bristol Pavilion at Gloucestershire's home cricket ground in Bristol.^8^ The artwork is a re-cast of an original created in 1888, which is on display at Lord's Cricket Ground Museum, under the ownership of the MCC.

## Medical retirement and a move to London

Twenty years after qualifying and successfully combining all that time with his medical practice in Bristol and first-class cricket, he ended his association with both England and Gloucestershire in 1899, as well as retiring from all medical work.

His abrupt departure surprised many and the reasons were not totally clear. It seems likely that his continued arrogant and autocratic style caused him to fall out with Gloucestershire County Cricket Club. However, a reduction in his medical work, caused by a restructure of districts, may also have contributed. He moved to Mottingham, a south-east London suburb, where Grace began the last phase of his career when he joined the newly formed London County Cricket Club in 1900 as player manager, where he continued to play first-class matches until the demise of this club in 1904.

His cricket appearances dwindled over the next four seasons. Grace made his final first-class appearance in April 1908 for the Gentlemen of England v Surrey, at The Oval. Overall, WG Grace, known by many as ‘the father of cricket’, had a glittering cricketing career, playing first-class cricket for 44 seasons from 1865 to 1908, during which he had captained England and Gloucestershire, arguably with his medical career residing in the shadows. A blue plaque was erected by the London County Council in 1963 on his previous residence in Mottingham;^9^ this was moved locally when the building was demolished.

**Figure 5.**
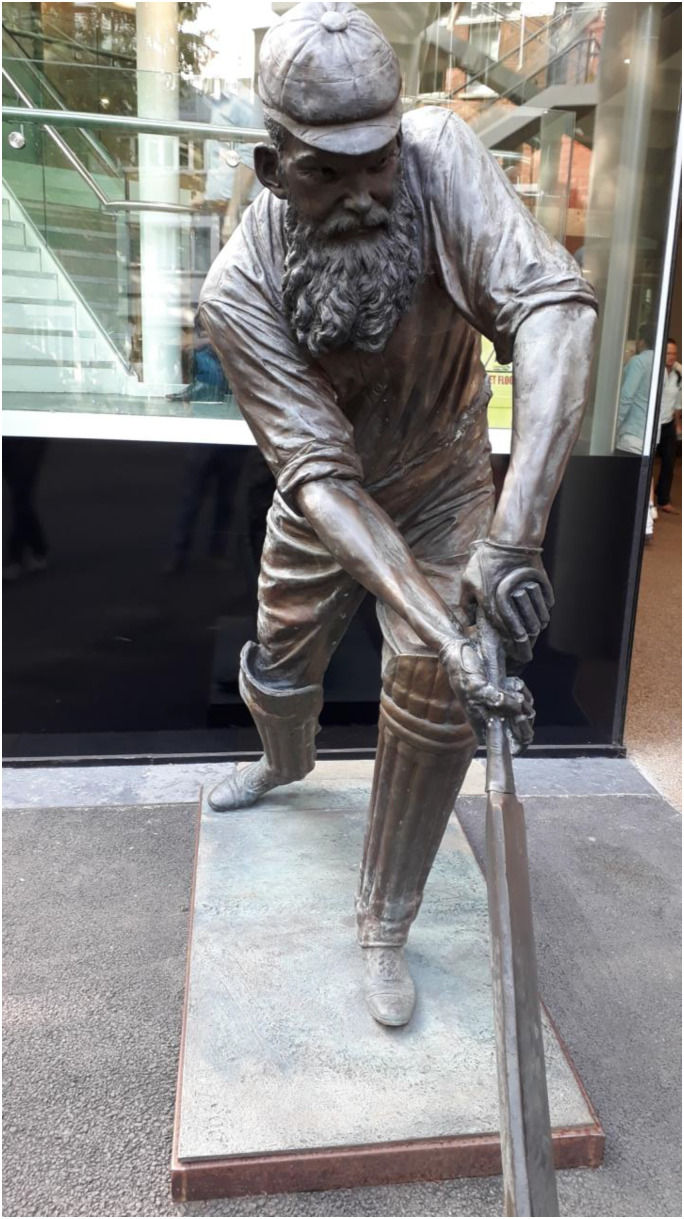
Statue of WG Grace at Lord's cricket ground.

Not surprisingly, Grace is commemorated at Lord's Cricket Ground ‘the home of cricket’. The WG Grace Memorial Gates, are two pairs of gates at Lord's in tribute to him, designed by architect Sir Herbert Baker, erected in 1923. Inside the ground, he is also commemorated with a bronze, full-length statue which was erected there in 2000, sculpted by the Australian-based artist, Louis Lamen (Figure 5).

He died from a cerebral haemorrhage in 1915 and is buried in the family grave at Beckenham, Kent.

Undoubtedly, Grace increased the popularity of cricket and he was a larger-than-life character. He was an extremely talented and competitive player, as well as one of the most controversial, on account of his gamesmanship and moneymaking. The range of memorials to him – wall plaques, murals, statues, public houses – reflects his widespread appeal and long-lasting legacy.
